# A case of endovascular treatment for pseudoaneurysm of the popliteal artery that developed 3 months after stenting

**DOI:** 10.1016/j.radcr.2025.11.086

**Published:** 2025-12-20

**Authors:** Takahiro Otsuka, Hideki Wada, Jun Shitara, Hirohisa Endo, Manabu Ogita, Satoru Suwa, Tohru Minamino

**Affiliations:** a Department of Cardiovascular Medicine, Juntendo University Shizuoka Hospital, Shizuoka, Japan; b Department of Cardiovascular Medicine and Biology, Juntendo University Graduate School of Medicine, Tokyo, Japan

**Keywords:** Endovascular treatment, Pseudoaneurysm, Popliteal artery, Stent graft

## Abstract

Pseudoaneurysms in lower limb arteries are rare, but can occur after endovascular procedures. We report the case of an 80-year-old man with a chronic limb-threatening ischemia who developed pseudoaneurysm at the site of a stent placed in the left popliteal artery 3 months earlier. Because of the severe pain and risk of aneurysm rupture, intervention was considered necessary. Considering the frailty of the patient, surgical treatment would have likely been difficult and endovascular treatment was therefore planned. A stent graft was placed, blocking blood flow to the pseudoaneurysm. This case demonstrates the effectiveness of stent grafts in managing post-endovascular pseudoaneurysm.

## Introduction

Lower extremity arterial disease is 1 manifestation of systemic atherosclerosis and is associated with significant morbidity and mortality [1,2]. Although endovascular therapy (EVT) has become a useful treatment option for popliteal artery diseases, the outcomes are not always acceptable [3]. Pseudoaneurysm can arise after a patient experiences complications injuring the vascular system, resulting in blood flow between the tunica media and tunica adventitia [4]. This in turn leads to the development of hematoma and compression of surrounding tissues. Pseudoaneurysms of the popliteal artery are rare and reported causes have involved complications from fractures, surgical interventions, and trauma and idiopathic cases. However, late aneurysm formation after EVT in the popliteal artery is very rare.

We report a case in which pseudoaneurysm developed at the site of a popliteal artery stent placed 3 months earlier. Treatment was provided using a stent graft. We also discuss the rationale for our treatment choice with reference to similar cases in the literature.

## Case report

**Fig. 1:**
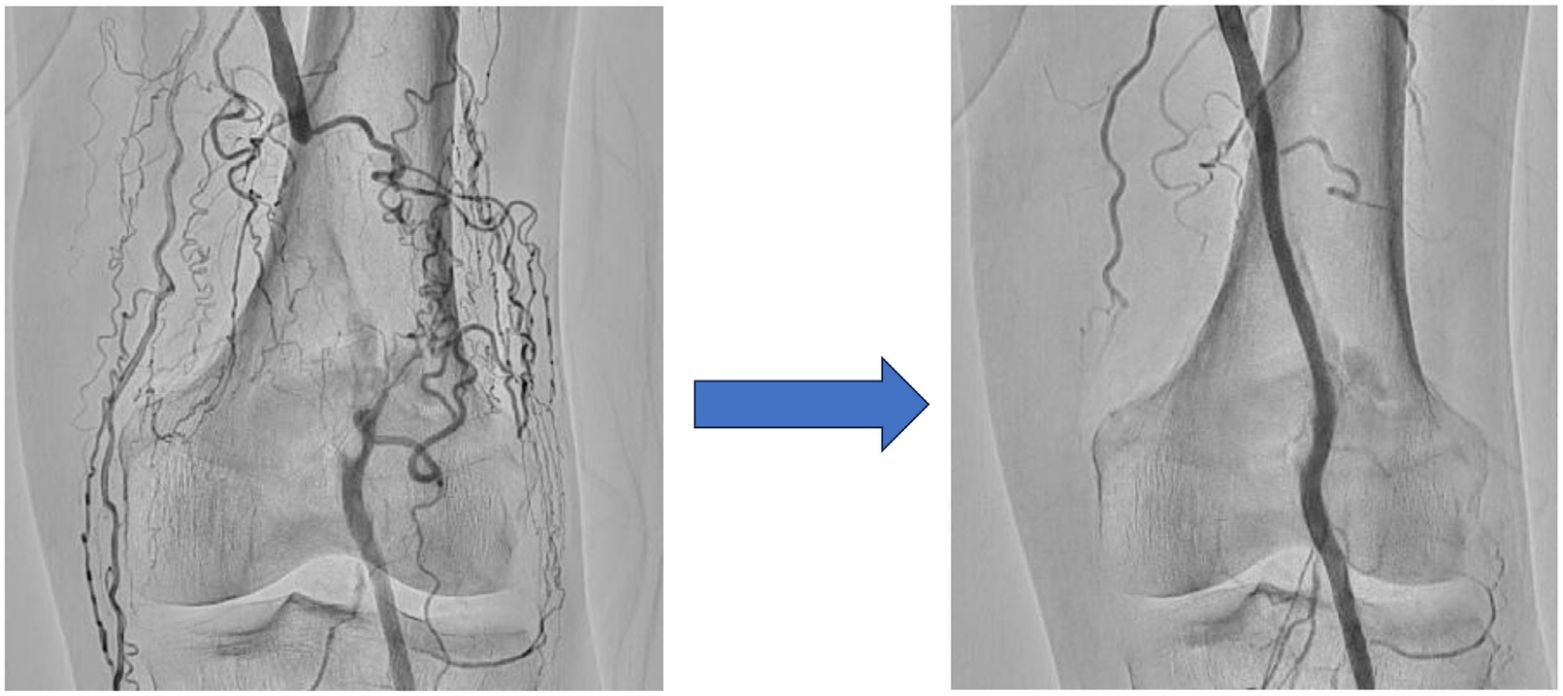
Endovascular treatment performed on the left popliteal artery 3 months before this presentation A previously treated severe calcified lesion in the left popliteal artery was found to be occluding the vessel (left panel). Endovascular treatment was therefore performed and a wire-interwoven stent (Supera peripheral stent, 5.5 × 80 mm) was implanted 3 months prior to this visit (right panel).

An 80-year-old man with a history of type 2 diabetes mellitus, dyslipidemia, hypertension, and coronary artery bypass grafting visited our hospital due to pain and swelling from the left lower back of the thigh to the back of the knee and an ulcer on the right foot. He had received several EVTs to address chronic limb-threatening ischemia for ulcers and rest pain in both legs. The patient had stenosis with severe calcification from the distal left superficial femoral artery to the popliteal artery, and had undergone a third EVT for the same lesion 3 months earlier. In that treatment, a wire-interwoven stent (Supera peripheral stent, 5.5 × 80 mm; Abbott Vascular Corporation, Chicago, IL, USA) was placed in addition to balloon angioplasty due to repeated restenosis (Fig. 1).

**Fig. 2:**
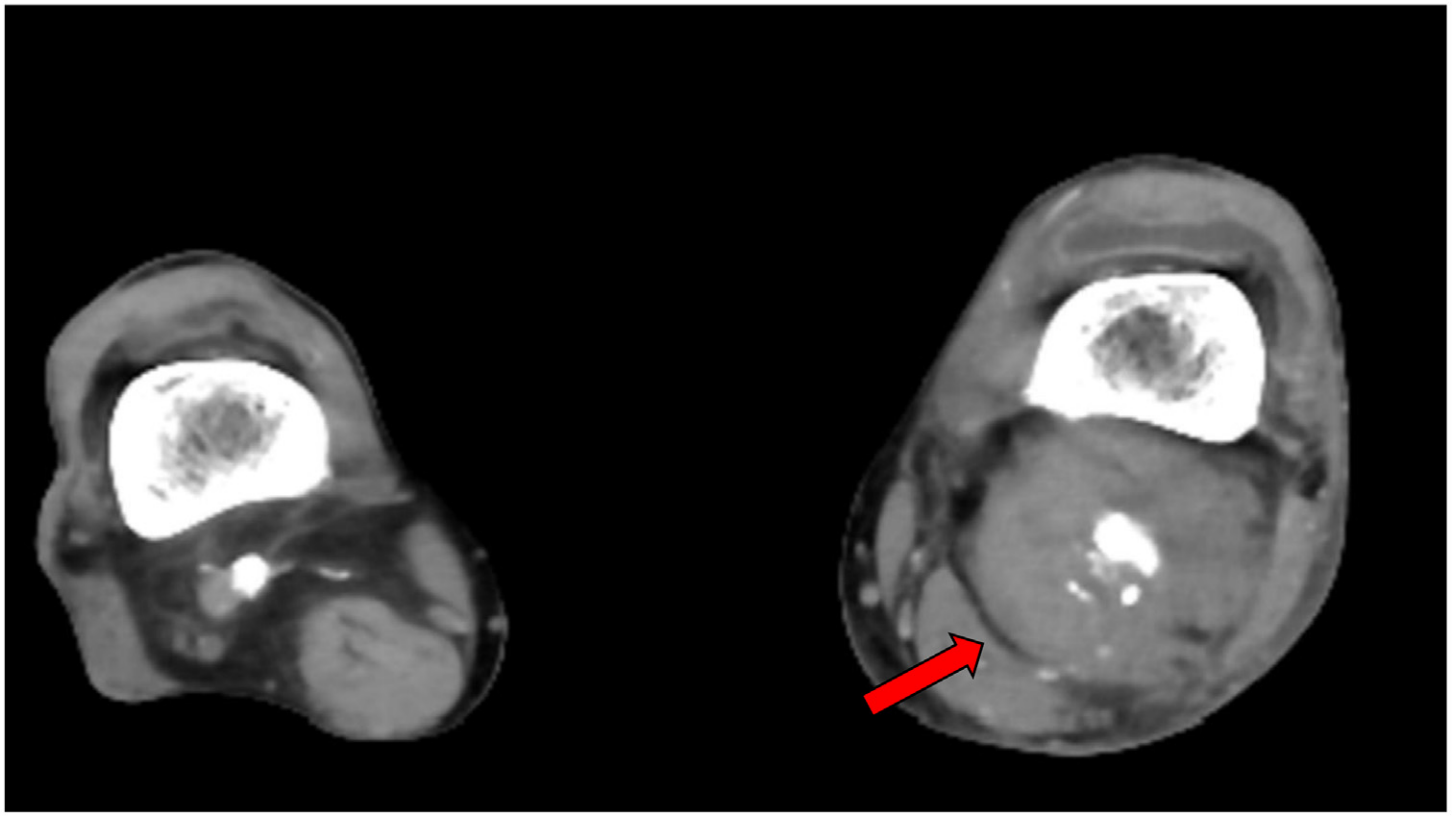
Popliteal artery imaged by contrast-enhanced computed tomography. Contrast-enhanced computed tomography reveals formation of a pseudoaneurysm with a maximum dimension of 54 × 55 mm in the left popliteal artery (red arrow).

**Fig. 3:**
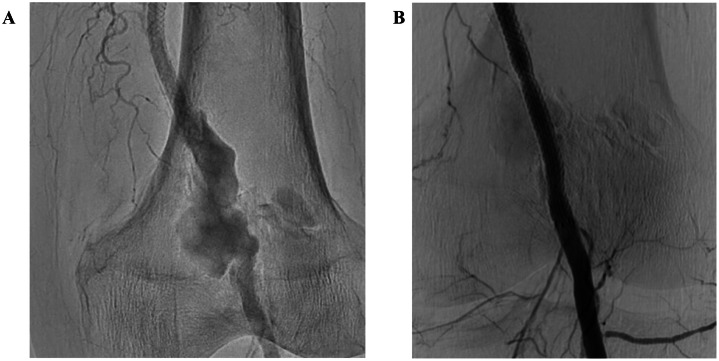
Angiographic findings. A. Angiographic findings of the popliteal artery pseudoaneurysm; B. Final angiography after stent graft implantation (A) Selective angiography shows aneurysm formation around the stent placed 3 months earlier. (B) After placement of the stent graft (VIABHN, 6.0 × 100 mm), blood flow to the pseudoaneurysm was confirmed to have ceased.

At the time of this visit, he was admitted to the plastic surgery department for treatment of an ulcer on the right foot. However, symptoms did not improve and he was referred to the department of cardiovascular medicine. The swollen area from the lower left thigh to the dorsal aspect of the knee was pulsatile, so we performed contrast-enhanced computed tomography, which revealed pseudoaneurysm with a maximum dimension of 54 × 55 mm at the popliteal artery (Fig. 2). Angiography performed later also showed aneurysm formation around the stent placed 3 months before (Fig. 3A). Because of the severe pain and risk of aneurysm rupture, intervention was considered necessary. Surgical resection of the aneurysm was an option, but considering the frailty of the patient, EVT using a stent graft was planned.

After puncturing the left common femoral artery, a guiding sheath was inserted and a guide wire was advanced to the distal rather than the lesion. We first examined the aneurysm lesion using an intravascular ultrasound device. A stent graft (VIABHN, 6.0 × 100 mm; Gore, Flagstaff, AZ, USA) was then implanted to cover the entire aneurysm lesion and post-dilatation was performed using a 6.0 × 40 mm balloon. Angiography and intravascular ultrasound confirmed excellent apposition of the stent graft and total exclusion of the pseudoaneurysms (Fig. 3B). After EVT, the patient reported rapid resolution of pain and swelling of the left leg gradually decreased.

## Discussion

Popliteal artery pseudoaneurysms are rare clinical conditions mainly linked to damage to the arterial wall, and are seen most often among young men. The literature has mentioned that the incidence of popliteal artery pseudoaneurysm ranges from 0.03% to 0.17%, representing around 4% of all popliteal artery aneurysms [5]. The pathology has also been reported to occur following local trauma [6], bony deformation [5], or iatrogenic injuries [7]. Here, we presented the case of an elderly man n with a large popliteal artery pseudoaneurysm after multiple EVTs. Some reports have shown that fracture of a self-expandable stent in the femoropopliteal artery caused pseudoaneurysm [8,9]. Stent fracture was not observed in the present case, but balloon dilatation or stenting might have damaged the vessel because the lesion was severely calcified.

Several options are available for the management of popliteal pseudoaneurysm, including open surgery, EVT or observation. Open surgical repair is considered the first choice for a large popliteal artery. EVTs using a covered stent or coiling may not provide reliable treatment for large aneurysms, and surgical treatment is preferred when possible. However, surgical repair requires the patient to be in a condition capable of withstanding general anesthesia, which may be difficult in frail patients. Endovascular repair has been shown to be safe and efficient for treating popliteal pseudoaneurysm, resulting in reduced morbidity and shorter duration of hospitalization. Rief et al. [7] reported a similar case involving a frail, bedridden, 90-year-old woman treated with a stent graft due to the difficulty of performing general anesthesia or surgical intervention. This approach was selected in that case as a minimally invasive and effective treatment for the pseudoaneurysm, taking into account the condition of the patient. On the other hand, the long-term patency issues and the risk of occlusion by thrombus in treatments with covered stents should also be considered. In addition, complete suppression of endoleak might be difficult when the pseudoaneurysm is large. In the present case, we were fortunate to be able to eliminate any endoleak by adding post-dilation after stent graft implantation.

## Conclusion

We achieved successful management of a post-endovascular pseudoaneurysm of the popliteal artery using a stent graft. Stent grafting proved to be an effective and less invasive treatment option, providing symptom relief and reducing aneurysm-related complications.

## Patient consent

The patient provided written informed consent for the publication of this case report, including all accompanying images and clinical information.
